# Folate Receptor β (FRβ) Expression in Tissue-Resident and Tumor-Associated Macrophages Associates with and Depends on the Expression of PU.1

**DOI:** 10.3390/cells9061445

**Published:** 2020-06-10

**Authors:** Rafael Samaniego, Ángeles Domínguez-Soto, Manohar Ratnam, Takami Matsuyama, Paloma Sánchez-Mateos, Ángel L. Corbí, Amaya Puig-Kröger

**Affiliations:** 1 Unidad de Microscopia Confocal, Instituto de Investigación Sanitaria Gregorio Marañón, Hospital General Universitario Gregorio Marañón, 28007 Madrid, Spain; 2 Centro de Investigaciones Biológicas, CSIC, 28007 Madrid, Spain; acorbi@cib.csic.es; 3 Barbara Ann Karmanos Cancer Institute and Department of Oncology, Wayne State University, Detroit, MI 48202, USA; ratnamm@karmanos.org; 4 Department of Immunology, Graduate School of Medical and Dental Sciences, Kagoshima University, Kagoshima 890-8544, Japan; matuyama@m.kufm.Kagoshima-u.ac.jp; 5 Laboratorio de Inmuno-Oncología, Instituto de Investigación Sanitaria Gregorio Marañón, Hospital General Universitario Gregorio Marañón, 28007 Madrid, Spain; paloma.sanchezmateos@salud.madrid.org; 6 Unidad de InmunoMetabolismo e Inflamación, Instituto de Investigación Sanitaria Gregorio Marañón, Hospital General Universitario Gregorio Marañón, 28007 Madrid, Spain; amaya.puig@iisgm.com

**Keywords:** macrophages, inflammation, rheumatoid arthritis, tumor-associated macrophages, folate receptor

## Abstract

As macrophages exhibit a huge functional plasticity under homeostasis and pathological conditions, they have become a therapeutic target for chronic inflammatory diseases. Hence, the identification of macrophage subset-specific markers is a requisite for the development of macrophage-directed therapeutic interventions. In this regard, the macrophage-specific Folate Receptor β (FRβ, encoded by the *FOLR2* gene) has been already validated as a target for molecular delivery in cancer as well as in macrophage-targeting therapeutic strategies for chronic inflammatory pathologies. We now show that the transcriptome of human macrophages from healthy and inflamed tissues (tumor; rheumatoid arthritis, RA) share a significant over-representation of the “anti-inflammatory gene set”, which defines the gene profile of M-CSF-dependent IL-10-producing human macrophages (M-MØ). More specifically, *FOLR2* expression has been found to strongly correlate with the expression of M-MØ-specific genes in tissue-resident macrophages, tumor-associated macrophages (TAM) and macrophages from inflamed synovium, and also correlates with the presence of the PU.1 transcription factor. In fact, PU.1-binding elements are found upstream of the first exon of *FOLR2* and most M-MØ-specific- and TAM-specific genes. The functional relevance of PU.1 binding was demonstrated through analysis of the proximal regulatory region of the *FOLR2* gene, whose activity was dependent on a cluster of PU.1-binding sequences. Further, siRNA-mediated knockdown established the importance of PU.1 for *FOLR2* gene expression in myeloid cells. Therefore, we provide evidence that FRβ marks tissue-resident macrophages as well as macrophages within inflamed tissues, and its expression is dependent on PU.1.

## 1. Introduction

Macrophages are phagocytic cells present in all tissues, whose huge functional plasticity allows them to drive promotion and resolution of inflammatory responses. Regardless of their origin, tissue-resident macrophages perform essential functions for maintenance of homeostasis [1], and usually exhibit more reparative and anti-inflammatory tasks than newly recruited blood-borne tissue-infiltrating macrophages [2] during inflammatory responses in numerous tissues [3,4,5,6,7,8,9]. As a representative example, tissue-resident macrophages in the lining layer of inflamed joints express higher levels of IL-10 than infiltrating macrophages during rheumatoid arthritis [10,11]. Macrophage Colony-Stimulating Factor (M-CSF), constitutively present in serum, promotes the differentiation and survival of tissue-resident and monocyte-derived macrophages (M-MØ) [12,13,14,15], and drives the acquisition of their anti-inflammatory and immunosuppressive functions (IL10^high^ TNF^low^ IL23^low^ IL6^low^) [16,17,18,19,20]. As a representative example, M-CSF determines the development and maturation of Kupffer cells [21], which have been implicated in both immunogenic and tolerogenic functions [22,23]. M-CSF triggers the acquisition of a characteristic gene expression profile that includes an “anti-inflammatory gene set” [19,24,25,26,27], whose presence characterizes human-tissue-resident macrophages [27,28,29,30] as well as tumor-associated macrophages (TAM) in vivo [16]. In fact, targeting the M-CSF/M-CSF receptor axis has been shown to reduce the presence of anti-inflammatory TAM in various tumors [31,32,33].

The “anti-inflammatory gene set” includes 170 genes whose expression is associated with the potent production of IL-10 after M-MØ stimulation. The expression of the “anti-inflammatory gene set” is critically dependent on the MAFB transcription factor in vitro [34,35] and might be also under the control of MAF in vivo [36,37]. The *FOLR2* gene is a member of the “anti-inflammatory gene set” that codes for the Folate Receptor β, a GPI-linked cell surface receptor with a high affinity for binding of folic acid (vitamin B9), whose physiologically reduced form (5-methyltetrahydrofolate) is a co-factor in one-carbon transfer reactions required for DNA and RNA synthesis, epigenetic processes, cellular proliferation and survival [38]. FRβ is a member of a family of reduced folate and folic acid receptors that also includes FRα, FRδ and FRγ, which differ in their respective cellular distribution and ligand selectivity [39]. While FRα is primarily expressed on the apical surface of epithelial cells and various tumors of epithelial origin [39,40,41], FRδ marks regulatory T cells and oocytes [42], and FRβ appears to be myeloid-restricted [43,44,45], although the molecular basis for its tissue-restricted expression remains unknown.

Macrophage reprogramming has been proposed as a therapeutic strategy for chronic inflammatory diseases [46]. Consequently, the identification of macrophage subset-specific markers is a requisite for the development of macrophage-directed therapeutic interventions for human pathologies. Because of its high affinity for folate binding and endocytosis, FRβ has been successfully used as a molecular target in therapeutic strategies for drug delivery and immune recognition in cancer and inflammatory pathologies [47,48]. In the present manuscript, we explore expression of *FOLR2*-encoded FRβ by tissue-resident macrophages in non-inflamed tissues and in TAM of various origins, and its correlation with the presence of genes commonly associated with the anti-inflammatory capacity of macrophages. Further, we investigate the dependence of the macrophage-specific expression of *FOLR2* on the ETS-domain transcription factor PU.1, which is essential for terminal myeloid cell differentiation [49,50] and control of expression of the M-CSF receptor [51]. Our results indicate that FRβ is a useful marker for tissue-resident macrophages and macrophages within inflamed tissues, and that its expression correlates with and depends on the expression of the PU.1 transcription factor.

## 2. Materials and Methods

### 2.1. Cell Culture and Flow Cytometry

The human cell lines K562, THP-1 and HeLa were obtained from the Centro de Investigaciones Biológicas Cell line repository. The cell lines K562 and THP-1 were cultured in RPMI supplemented with 10% fetal calf serum (FCS) at 37 °C in a humidified atmosphere with 5% CO_2_. HeLa cells were maintained in DMEM supplemented with 10% FCS. Monocyte-derived macrophages M-MØ were generated in the presence of M-CSF, as previously described [34]. Phenotypic analysis was carried out by indirect immunofluorescence using a mouse anti-human-FRβ antibody [52] and using isotype-matched monoclonal antibody as a negative control. Folate-FITC endocytosis assays were done as previously reported [45].

### 2.2. Transfections, Plasmids, and Site-Directed Mutagenesis

In reporter gene experiments, the FOLR2-based reporter gene construct pFOLR2-200Luc [19] was transfected in HeLa cells using Superfect (Qiagen, Hilden, Germany) and in THP-1 cells through the use of the Cell Line Nucleofector Kit V (Amaxa, Cologne, Germany). The amount of DNA in each transfection was normalized by using the corresponding insertless expression vector (CMV-Ø) as a carrier. Each transfection experiment was performed at least three times with different DNA preparations. Transfection efficiencies were normalized by co-transfection with the pCMV-ßgal plasmid, and β-galactosidase levels were determined using the Galacto-Light kit (Tropix, Bedford, MA 01730, USA). The PU.1 expression plasmid has been previously described [53]. Site-directed mutagenesis on the pFOLR2-200Luc promoter construct was done using the QuikChange System (Stratagene, La Jolla, CA 92037, USA). For mutation of the PU.1-64 and PU.1-60 elements, the oligonucleotides PU.1-64mutS (5′CCTTGAAGAGGGTGGGGTGACGATCCGATGGAAGAGAGGAAGGAGAATAG-3′) and PU.1-64mutAS (5′-CTATTCTCCTTCCTCTCTTCCATCGGATCGTCACCCCACCCTCTTCAAGG-3′) were used, and the resulting plasmid was termed pFOLR2-200PUmut2Luc. Generation of the pFOLR2-200PUmut4Luc plasmid, where the PU.1-binding sequences PU.1-64, PU.1-60, PU.1-55 and PU.1-47 are mutated, was accomplished by site-directed mutagenesis on the pFOLR2-200PUmut3Luc plasmid, using the oligonucleotides PU.1-47S (5′-GGTGACGATCCGATCGATGACTCGATGGAGAATAGCTAAGTAGGG-3′) and PU.1-47AS (5′-CCCTACTTAGCTATTCTCCATCGAGTCATCGATCGGATCGTCACC-3′). DNA constructs and mutations were confirmed by DNA sequencing. DNA sequencing was performed at the Genomics Unit of the Hospital General Universitario Gregorio Marañón.

### 2.3. Melanoma Xenograft Model

Immunodeficient NOD-*scid*-IL2Rg^null^ (NSG) mice (The Jackson Laboratory, Bar Harbor, ME 04609, USA) were maintained under specific pathogen-free conditions. Male mice (4–6 weeks of age) were subcutaneously inoculated with 10^6^ BLM melanoma cells. When tumors reached approximately 1 cm in width (approximately at day 14th), mice were euthanized and tumors were resected and frozen for histologic analyses. This procedure was approved by the IiSGM animal care/use and Comunidad de Madrid committees (PROEX-084/18).

### 2.4. Confocal Microscopy and Immunohistochemistry

Normal skin samples were obtained from abdominoplasty. Normal colon and muscle samples were localized adjacent to tumor and obtained from colon adenocarcinoma and melanoma patients. Informed consent was obtained, and all the procedures were performed following Medical Ethics Committee (Hospital General Universitario Gregorio Marañón) guidance. Thick sections (4 μm in depth) of cryopreserved tissue were first blocked for 10 min with 1% human immunoglobulins and then incubated for 1 h with either a mouse monoclonal antibody against human FRβ [52], a rat monoclonal antibody against murine FRβ [48], an anti-CD163 monoclonal antibody (clone Ber-Mac3, MBL International Corp., Woburn, MA 01801, USA), an anti-Von Willebrand factor (rabbit polyclonal, Dako, Santa Clara, CA 95051, USA), an anti-F4/80 (clone BM8, labeled with Alexa Fluor 647, Biolegend, San Diego, CA 92121, USA), or isotype-matched control antibodies. All primary antibodies were used at 1–5 μg/mL, followed by incubation with Cy3-labeled anti-mouse and Cy5-labeled anti-rabbit secondary antibodies. Tissues were imaged with the 20X PL-APO NA 0.7 immersion objective of a confocal scanning inverted AOBS/SP2-microscope (Leica Microsystems, Wetzlar, 35578 Germany). Image processing was assessed with the Leica Confocal Software LCS-15.37. Tissue microarrays (SuperBio Chips AC1 Human, Clinisciences, 92000 Nanterre, France) were processed according to manufacturer´s recommendations and stained with a mouse monoclonal antibody against CD68 (PG-M1; Dako, Santa Clara, CA 95051, USA) and a rabbit polyclonal antibody against FRβ [54].

### 2.5. Quantitative Real Time RT-PCR

Oligonucleotides for selected genes were designed according to the Roche software for quantitative real-time PCR, and RNA was amplified using the Universal Human Probe Roche Library (Roche Diagnostics, Indianapolis, IN 46256, USA). Assays were made in triplicate and results normalized according to the expression levels of *GAPDH*. In all cases, the results were expressed using the ΔΔCT method for quantitation.

### 2.6. siRNA-Mediated Knockdown

THP-1 cells (2 × 10^6^ cells) were nucleofected with 3 μg of siRNA for PU.1 (sc-36330 PU.1 siRNA gene silencer; Santa Cruz Biotechnology, Dallas, Texas 75220 USA) or a negative control siRNA (sc-37007 Control siRNA-A; Santa Cruz Biotechnology, Dallas, Texas 75220 USA) using the Cell Line Nucleofector Kit V (Amaxa, Cologne, Germany). After nucleofection, cells were kept in culture for 24 h, and one-fifth of the cells were lysed and subjected to Western Blot for PU.1 detection. Total RNA was isolated from the remaining nucleofected cells and subjected to real time-PCR.

### 2.7. Bioinformatic Analysis

The genes selectively expressed by monocytes and macrophages from human gut were obtained from [55] and used to identify those genes contained within the “Pro-inflammatory gene set” and “Anti-inflammatory gene set” previously defined [19,29]. A list of genes specifically expressed by macrophages within melanoma [56] and head and neck squamous carcinoma [57] was derived using Cibersortx [58], and their expression in breast carcinoma determined using the METABRIC (Molecular Taxonomy of Breast Cancer International Consortium) study cohort [59,60] on the cBioPortal for Cancer Genomics [61] and using the TIMER resource [62,63,64] on data generated by The Cancer Genome Atlas Program Research Network [65]. Identification of genes co-expressed with *FOLR2* in various tissues was done using Genevestigator® [66]. Gene ontology analysis of the defined gene sets was performed using the online tool ENRICHR [67,68]. Chip-seq data were derived from the Cistrome data browser [69] and processed using the WashU Epigenome Browser [70].

### 2.8. Statistical Analysis

Statistical analysis was performed using a paired Student´s *t*-test and a *p* value < 0.05 was considered significant.

## 3. Results

### 3.1. Folate Receptor Beta (FRβ) is Co-Expressed with other Genes of the “Anti-Inflammatory Gene Set” and Marks Human Tissue-Resident Macrophages

**Figure 1:**
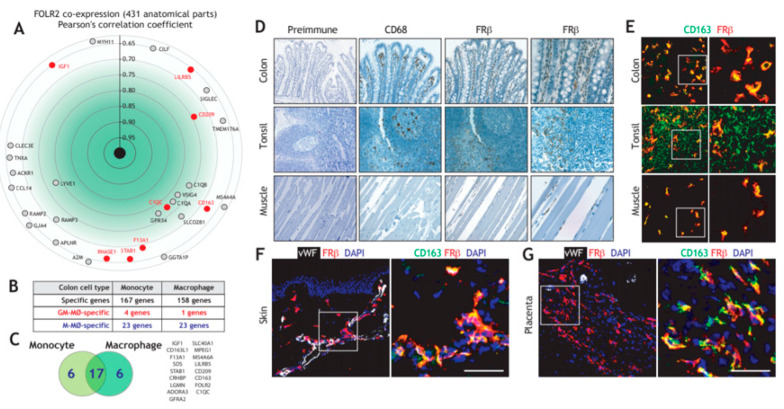
Expression of *FOLR2* and FRβ in human tissue-resident macrophages. (**A**) Identification of genes of the “anti-inflammatory gene set” whose expression most significantly correlate with *FOLR2* expression, using Genevestigator^®^ [66]. Pearson´s correlation coefficients are indicated in each case. (**B**) Overlapping of M-MØ-specific and GM-MØ-specific genes within the lists of monocyte- or macrophage-restricted genes in human colon samples [55]. (**C**) Identification of M-MØ-specific genes specific within the lists of monocyte- and macrophage-restricted genes in human colon samples. (**D**) FRβ expression in distinct normal tissues. Light microscopy images of the macrophage marker CD68 and FRβ staining in colon, tonsil and skeletal muscle (magnification, ×20). The right panel indicates a higher magnification (magnification ×40) for FRβ staining. Left, staining yielded by normal rabbit serum, used as a control (pre-immune); (**E**) Co-expression of FRβ and CD163 in colon, tonsil and skeletal muscle. Confocal images of human colon, tonsil and skeletal muscle tissue sections, as determined by double immunofluorescence analysis of CD163 (green) and FRβ (red) expression. Yellow color indicates the FRβ/CD163 merged colocalizing areas. Right panels show a magnification of a FRβ/CD163 colocalizing area (marked in a box in left panels); (**F**–**G**) Co-expression of FRβ and CD163 in human dermis (**F**) and placenta (**G**). Confocal images of human tissue sections, as determined by triple immunofluorescence analysis of Von Willebrand factor (white), CD163 (green) and FRβ (red) expression. Yellow color indicates the FRβ/CD163 merged co-localizing areas. The area marked by boxes is shown at a higher magnification in the right panel. Nuclei are counterstained with DAPI. Scale bars: 50 μm.

We have previously reported the existence of transcriptional overlaps between the variety of macrophage subsets that make up Tumor-Associated Macrophages (TAM) and M-CSF dependent macrophages, as both are enriched in the expression of the 170-gene “anti-inflammatory gene set” [19,29], which includes the FRβ-encoding *FOLR2* gene [45]. Assessment of the genes significantly co-expressed with *FOLR2* in 431 anatomical locations identified various genes of the “anti-inflammatory gene set”, including *CD209*, *C1QC*, *CD163*, *LILRB5*, *F13A1*, *STAB1*, *RNASE1* and *IGF1*, with Pearson´s correlation coefficients ranging from 0.77 to 0.65 (Figure 1A). In line with these findings, analysis of monocytes or macrophages from human colon, whose transcriptomes have been extensively analyzed [55], also revealed an enrichment of the “anti-inflammatory gene set” (Figure 1B), also including the expression of *CD209*, *C1QC*, *CD163*, *LILRB5*, *F13A1* and *IGF1* from the “anti-inflammatory gene set” (Figure 1C). Given these results, *FOLR2*-encoded FRβ expression was evaluated in colon and other tissue macrophages under homeostatic conditions. In agreement with the transcriptional data, and using tissue arrays, an FRβ-specific antiserum [54] stained numerous cells in the lamina propria of the colon, where CD68+ macrophages were also detected (Figure 1D). Besides, FRβ+ cells were found in the paracortical area of the tonsil and, to a lesser extent, in skeletal muscle (Figure 1D). Further, multicolor immunofluorescence revealed that FRβ is co-expressed with the hemoglobin/haptoglobin scavenger receptor CD163 in lamina propria macrophages, as well as in tonsil and in skeletal muscle, thus indicating that FRβ marks tissue-resident macrophages (Figure 1E). In addition, FRβ was co-expressed with CD163 in the dermis (Figure 1F), where most FRβ+/CD163+ macrophages exhibited a perivascular distribution (Figure 1F), as well as in placenta [71] (Figure 1G). Therefore, FRβ is broadly expressed in vivo by tissue-resident macrophages, where its expression positively correlates with the macrophage marker CD163 and other genes of the “anti-inflammatory gene set”.

### 3.2. FOLR2/FRβ Expression Marks Human Tumor-Associated Macrophages and Correlates with the Expression of CD163 and Regulators of Macrophage Differentiation

**Figure 2:**
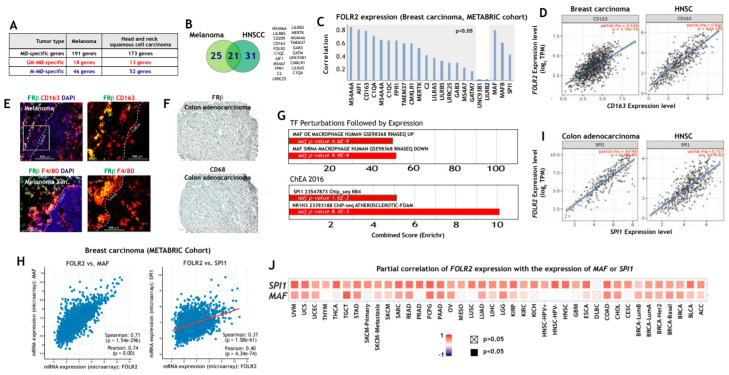
Expression of *FOLR2* and FRβ in human tumor-associated macrophages. (**A**) Overlapping of M-MØ-specific and GM-MØ-specific genes within the lists of macrophage-specific genes in human melanoma [57] or head and neck squamous carcinoma (HNSC) samples [58]. (**B**) Identification of M-MØ-specific genes specific within the lists of macrophage-specific genes in human melanoma [57] and head and neck squamous carcinoma samples [58]. (**C**) Correlation of the expression of the *FOLR2* gene and the expression of the 21-gene dataset shown in B. The correlation with the expression of MAF, MAFB and SPI1 is also shown. The shaded area indicates the significant positive correlations. (**D**) Correlation of the expression of the *FOLR2* gene with the expression of C*D163* in breast carcinoma and HNSC (TCGA Cohort). (**E**) Co-expression of FRβ and CD163 in human melanoma (upper panels) and a human melanoma xenograft (lower panels) using confocal microscopy after double immunofluorescence analysis of CD163 (red) and FRβ (green) expression. Yellow color indicates the FRβ/CD163 merged colocalizing areas. Right panels show a magnification of the FRβ/CD163 colocalizing area (marked in a box in left panels). (**F**) FRβ and CD68 expression in a sample of human colon adenocarcinoma. Light microscopy images of the macrophage marker CD68 and FRβ staining (magnification, ×20). (**G**) Enrichr analysis [67,68] of the genes indicated in panel B. (**H**) Correlation of the expression of the *FOLR2* and *SPI1* genes in colon adenocarcinoma and HNSC (TCGA Cohort). (**I**) Correlation of the expression of the *FOLR2* gene with *MAF* and *SPI1* expression in breast carcinoma (METABRIC Cohort). (**J**) Partial correlation of *FOLR2* expression with the expression of *MAF* or *SPI1* in the indicated tumors.

To determine whether the macrophage-restricted expression of FRβ also applies to pathological settings, we next assessed *FOLR2* expression in TAM from various tumor types. To this end, we initially searched for macrophage-specific gene expression in melanoma [57] and head and neck squamous cell carcinoma (HNSC) [58] (Figure 2A) and identified a set of 21 M-MØ-specific genes whose expression is also restricted to melanoma and HNSC TAM (Figure 2B). Interestingly, *FOLR2*, *CD163*, *LILRB5*, *CD209* and *C1QC* were identified as genes of the “anti-inflammatory gene set”, whose expression is also seen in tissue-resident macrophages and tumor-associated macrophages (Figure 2B). Analysis of breast cancer transcriptomes (METABRIC cohort) also evidenced a very good correlation between the expression of *FOLR2* and those of genes of the “anti-inflammatory gene set”, which reached statistical significance in most cases (Figure 2C), and was highly significant for *CD163* (Pearson: 0.72; *p* = 1.71 × 10^−308^) and even CD68 (Pearson: 0.59; *p* = 1.25 × 10^−176^), another widely used marker for macrophage identification (Figure 2C). Further, a significant correlation was found between the expression of *FOLR2* and *CD163* using the TCGA cohorts for breast carcinoma, melanoma and HNSC (Figure 2D and not shown), whereas no correlation was seen between *FOLR2* and the epithelial-specific *EPCAM* gene in any of the analyzed tumors (data not shown). In fact, and at the protein level, FRβ expression was observed in CD163+ cells in melanoma (Figure 2E) and in areas enriched in CD68+ cells in colon adenocarcinoma (Figure 2F). Interestingly, and since FRβ+ macrophages are prominent in the tumor-invasive front of pancreatic cancer and associate with poor prognosis [72], it is worth noting that FRβ+ macrophages were mostly detected in the peritumoral area both in human melanoma and in a melanoma xenograft mouse model (Figure 2E). Altogether, this set of results indicates that *FOLR2* expression correlates with the expression of *CD163* and other macrophage-specific genes in TAM, and that FRβ expression in TAM overlaps with the expression of the commonly used macrophage markers CD163 and CD68.

To identify potential regulators of *FOLR2* gene expression, gene ontology analysis was done using Enrichr [64], and results revealed a positive enrichment of genes regulated by *MAF*, *SPI1* (PU.1) and *NR1H3* (LXRα) in the TAM-specific genes of the “anti-inflammatory gene set” (Figure 2G). Indeed, *FOLR2* expression was found to correlate with the expression of genes coding for transcription factors that determine macrophage differentiation and specification (*SPI1* and *MAF*) in breast carcinoma (METABRIC cohort, Figure 2H), thus suggesting their involvement in expression of the FRβ-encoding *FOLR2* gene. Further analysis of a large variety of tumor types using TIMER2.0 revealed that the positive correlation between *FOLR2* and *SPI1* expression was highest in colon adenocarcinoma, HNSC and sarcoma (Figure 2I), and that the *FOLR2*–*SPI1* correlation was more significant than the *FOLR2*–*MAF* correlation in almost every tumor type (Figure 2J). Conversely, no significant correlation was found between *FOLR2* or *SPI1* expression and the epithelial-specific *EPCAM* gene expression (data not shown). Altogether, these results established a link between the *FOLR2* gene and the expression of the PU.1-encoding *SPI1* gene in tumor-associated macrophages.

### 3.3. FOLR2/FRβ Expression also Marks Human Synovial Macrophages

**Figure 3:**
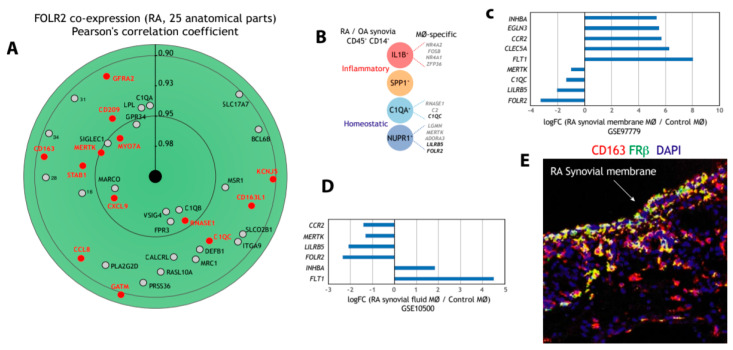
Expression of *FOLR2* and FRβ in human synovium. (**A**) Identification of genes of the “anti-inflammatory gene set” whose expression most significantly correlates with *FOLR2* expression in RA, using Genevestigator^®^ [66]. Pearson´s correlation coefficients are indicated in each case. (**B**) Expression of genes of the “anti-inflammatory gene set” in each of the four monocyte subsets defined in inflamed synovial tissue by scRNA-seq [69]. (**C**,**D**) Comparison of the expression of representative M-MØ-specific (“anti-inflammatory gene set”) and GM-MØ-specific genes in control and inflamed synovial membrane (**C**) and synovial fluid (**D**). (**E**) Co-expression of FRβ and CD163 in human synovial membrane from an RA patient using confocal microscopy after double immunofluorescence analysis of CD163 (red) and FRβ (green) expression. Yellow color indicates the FRβ/CD163 merged colocalizing areas.

Next, we address the macrophage-restricted expression of *FOLR2* in a pathology where macrophages preferentially exhibit a pro-inflammatory polarization, rheumatoid arthritis (RA). Initial assessment of *FOLR2* expression in RA indicated an extremely close correlation with the expression of the “anti-inflammatory gene set” (Figure 3A). In fact, 13 genes of the “anti-inflammatory gene set” were found within the 50 genes more closely correlating with *FOLR2* expression in RA (Figure 3A). Single-cell RNA sequencing (scRNA-seq) on samples from patients with rheumatoid arthritis (RA) or osteoarthritis (OA) has identified 18 unique cell populations in synovial tissue, including four transcriptionally different monocyte subsets [73]: IL1B+ pro-inflammatory monocytes, IFN-activated SPP1+ monocytes, NUPR1+ monocytes and C1QA+ monocytes (Figure 3B), with the latter two subsets under-represented in RA and thought to exert homeostatic functions [73]. Analysis of the four subsets revealed the presence of genes of the “anti-inflammatory gene set” in IL1B+, NUPR1+ and C1QA+ monocytes, and that the expression of *FOLR2* is a specific marker for the NUPR1+ monocyte subset (Figure 3B) [73]. Indeed, although the expression of *FOLR2* diminishes in macrophages from synovial membranes [74] (Figure 3C) and from synovial fluid [75] (Figure 3D) in RA, FRβ is still detectable in the lining layer of the synovial membrane of RA patients (Figure 3E).

### 3.4. Expression of FRβ in Myeloid Cells is Dependent on the PU.1 Transcription Factor

**Figure 4:**
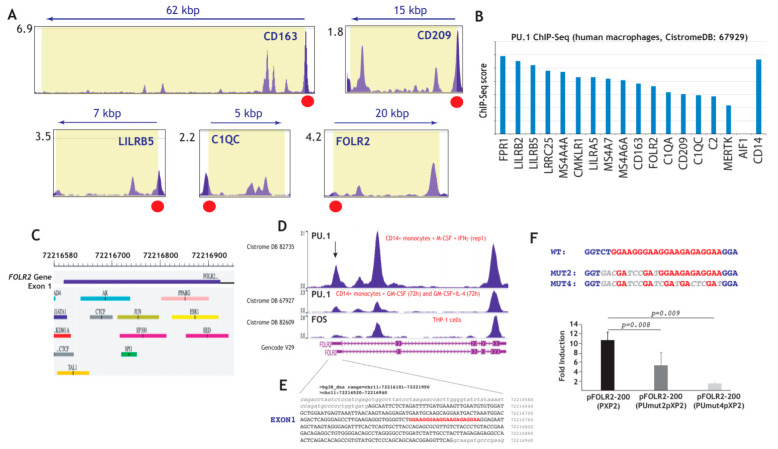
Structural and functional analysis of the *FOLR2* proximal regulatory region. (**A**) Identification of PU.1-binding sites immediately upstream of the first exon of the indicated genes (data obtained from CistromeDB 92249 [72]). (**B**) ChIP score of the indicated genes in the PU.1 ChIP-Seq experiment done on human macrophages (data obtained from CistromeDB 67929 [73]). (**C**) Schematic representation of the first exon of the *FOLR2* gene, with indication of predicted transcription factor binding sites [70]. (**D**) Schematic representation of the ChIP-Seq data on the *FOLR2* gene from the indicated experiments. (**E**) Nucleotide sequence of the first exon of the human *FOLR2* gene (uppercase) and flanking sequences (lowercase), with indication of the PU.1-binding elements. (**F**) HeLa cells were transfected with the indicated FOLR2 promoter-based reporter plasmids in the presence of an empty vector or expression vector for PU.1. For each individual reporter construct, fold induction represents the luciferase activity yielded by PU.1 expression vector relative to the activity produced by an identical amount of empty CMV-0 plasmid. Luciferase activity was determined after 24 h. Data represent the mean ± standard deviation of five independent experiments using two different DNA preparations. The nucleotide sequence of the PU.1-binding elements in the WT and mutant constructs is indicated at the top.

To obtain support for the potential involvement of PU.1 in *FOLR2* gene expression, we revised the available ChIP-Seq information on the genes of the “anti-inflammatory gene set”, which had been found to be significantly expressed in tissue-resident macrophages (Figure 1) and TAM (Figure 2), and identified validated PU.1-binding sites immediately upstream of the first exon of most genes (Figure 4A and not shown) [76], including *FOLR2* as well as PU.1-binding sites within most genes of the “anti-inflammatory gene set” (Figure 4B) [77]. The presence of 3–4 major peaks in ChIP-seq data for PU.1 binding within the *FOLR2* gene [74,76,77], with one of them localized within exon 1, close to a potential FOS-binding site [78] (Figure 4C,D) and overlapping a sequence containing four evolutionary conserved potential Ets-binding sequences (5′-GGAAGGGAAGGAAGAGAGGAA-3′) [79,80] (Figure 4D,E), led us to address the control of *FOLR2* expression by PU.1. To analyze the functional significance of this cluster of PU.1-binding elements, we initially evaluated its contribution to the transcriptional activity of the *FOLR2* proximal promoter. In HeLa cells devoid of PU.1 [81,82], transfected with the pFOLR2-200Luc construct, which contains the fragment -214 to -34 and includes the PU.1-binding elements, overexpression of PU.1 resulted in 10-fold enhancement of the activity of the promoter (Figure 4F). Further, mutation of the two distal Ets elements (pFOLR2-200PUmut2pXP2, Figure 4F) reduced PU.1-dependent transactivation to 50% (*p =* 0.008), while mutation of the four Ets-sequences (pFOLR2-200PUmut4pXP2, Figure 4F) reduced PU.1 transactivation by 86% (*p =* 0.009), thus implying that PU.1 exerts a positive regulatory action on the FOLR2 proximal regulatory region through interaction with a cluster of Ets-cognate sequences within exon 1 of the *FOLR2* gene.

**Figure 5:**
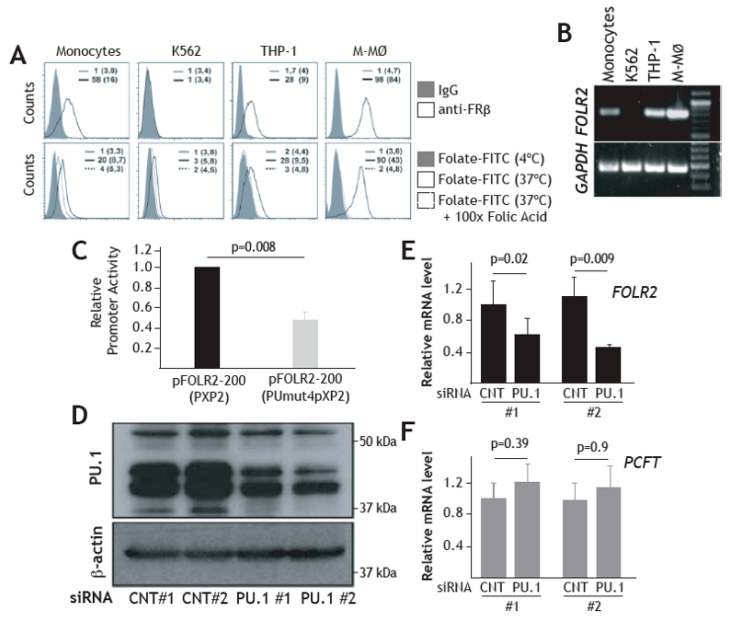
PU.1 enhances FOLR2 gene promoter activity and increases FOLR2 gene expression. (**A**) Upper histograms, FRβ expression (empty histograms) in monocytes, K-562, THP-1 and M-MØ macrophages, as determined by flow cytometry. Lower histograms, internalization of Folate-FITC by monocytes, THP-1, K-562 and M-MØ macrophages, in the absence (black line) or the presence (dotted line) of a 100-molar excess of folic acid. Filled histograms indicate cell autofluorescence. In each case, the percentage of marker-positive cells and the mean fluorescence intensity (in parenthesis) are indicated. The experiment was performed five times, and one of the experiments is shown. (**B**) Detection of *FOLR2* and *GAPDH* mRNA by RT-PCR on RNA from CD14+ peripheral blood monocytes, K-562, THP-1 and M-MØ macrophages. Molecular size markers were loaded in lane 5. (**C**) THP-1 cells were nucleofected with the indicated reporter plasmids and luciferase activity was determined after 24h. Promoter activity is expressed relative to the activity produced by the wild-type pFOLR2-200pXP2, arbitrarily set to 1, after normalization for transfection efficiency (*n* = 6). (**D**,**E**) siRNA-mediated knockdown of PU.1. THP-1 cells were nucleofected with either siRNA for PU.1 (siRNA PU.1) or a control siRNA (siRNA CNT). After 24 h, one third of the cells were lysed and subjected to western blot for PU.1 expression (**D**), and total RNA was isolated and *FOLR2* and *PCFT* mRNA were determined by quantitative RT-PCR (**E**). Results are expressed as relative mRNA levels (relative to *GAPDH* mRNA levels and the corresponding mRNA level in control #1 siRNA-nucleofected cells). The experiment was performed in duplicate, and both experiments are shown.

Having demonstrated a direct effect of PU.1 on the *FOLR2* proximal regulatory region, we then assayed the role of PU.1 on the activity of the *FOLR2* promoter in the FRβ-expressing human THP-1 myeloid cell line, where the receptor is expressed in a functional state (Figure 5A,B). As shown in Figure 5C, the pFOLR2-200PUmut4pXP2 construct exhibited significantly lower activity than the wild-type pFOLR2-200pXP2 construct (*p = 0.008*) in THP-1 cells, thus indicating that the activity of the *FOLR2* gene regulatory region in myeloid cells is partly dependent on the integrity of the cluster of PU-1-binding elements located within exon 1. To definitively prove the direct involvement of PU.1 on FRβ expression, *FOLR2* mRNA expression level was assessed after knocking down PU.1 expression in FRβ+ THP-1 cells. Nucleofection of a PU.1-specific siRNA in THP-1 cells reduced the expression of PU.1 by more than 50% (Figure 5D). More importantly, siRNA-mediated knockdown of PU.1 led to a significant down-modulation of *FOLR2* mRNA levels (*p* = 0.02 for experiment #1 and *p* = 0.009 for experiment #2) without affecting the expression of the functionally related *PCFT* gene (Figure 5E). Therefore, PU.1 regulates *FOLR2* gene expression in THP-1 cells, further confirming PU.1 dependence on the myeloid-restricted expression of the *FOLR2* gene.

## 4. Discussion

In the present manuscript, we show that the *FOLR2* gene is expressed by human CD163+ tissue-resident and tumor-associated macrophages (TAM) from various sources, and that its restricted cellular distribution is shared by a limited number of genes, including the commonly used macrophage-specific marker CD163. CD163 is a bona fide macrophage-specific marker [78] that, however, is expressed at higher levels in macrophages polarized toward the anti-inflammatory and reparative side [18,19], a property also shared by FRβ [45]. Indeed, *FOLR2* expression parallels that of *CD163* in tissue-resident macrophages, TAM from various tumor types and inflamed synovium. Therefore, FRβ can be considered a macrophage-specific marker, in line with previous reports on its expression in distinct macrophage subsets in human and mouse tissues [44,45,48,72,83,84]. Further stressing its cell-restricted expression, the expression of *CD163* and *FOLR2* in TAM significantly correlates with the presence of the PU.1 transcription factor. Thus, we have found that the PU.1 transcription factor, which is preferentially expressed in myeloid cells, enhances the transcriptional activity of the proximal regulatory region of the *FOLR2* gene and directly influences *FOLR2* gene expression. The demonstration of the PU.1-dependent expression of *FOLR2* is the first evidence of a transcription factor directly controlling *FOLR2* expression and suggests that PU.1 contributes to the myeloid-specific expression of FRβ.

Macrophage reprogramming now appears a feasible therapeutic strategy for chronic inflammatory diseases [46]. Accordingly, the identification of macrophage subset-specific markers is a requisite for the development of macrophage-directed therapeutic interventions for human pathologies. The identification of FRβ as a macrophage-specific marker in homeostatic and pathological states has relevant translational implications, because FRβ has already been used as a target for imaging and delivery of therapeutic agents in inflammation-related diseases like rheumatoid arthritis [83,84,85,86]. Therefore, it is tempting to hypothesize that FRβ might also be a useful tool for delivery of agents with ability to shift the macrophage polarization state. Such an approach would benefit from the constitutive FRβ recycling ability [87,88,89] as well as by its huge capacity to transfer ligands towards the macrophage endocytic machinery [89,90]. This strategy would be particularly well suited in the case of tumors, as FRβ is highly expressed in TAM (this report and [45]). TAM promotes malignancy by stimulating angiogenesis, tumor-cell migration and invasion, and TAM accrual correlates with a worse prognosis in numerous tumors (85). Thus, FRβ constitutes an ideal target for delivery of macrophage-repolarizing agents into TAM, and, in line with the results here presented, the characterization of the factors that regulate FRβ expression constitutes relevant information for the development of FRβ-based macrophage targeting strategies.

Besides the involvement of PU.1 in the myeloid expression of FRβ, bioinformatics analysis also indicates that FOLR2 gene expression closely correlates with the expression of the MAF transcription factor in TAM from various sources. In fact, *FOLR2* exhibits the highest level of correlation with *MAF* expression in breast carcinoma, a finding that agrees with the considerable decrease in *FOLR2* expression that is seen upon *MAF* knockdown in human macrophages [74]. However, ChIP-Seq has not provided evidence for any interaction of MAF with the *FOLR2* gene. Considering the ability of MAF to heterodimerize with members of the JUN/FOS family of transcription factors [91], and given the existence of FOS-binding sites in exon 1 [78] and additional AP-1-binding elements nearby [79], it is conceivable that MAF might indirectly affect *FOLR2* expression by altering the levels of available JUN/FOS family proteins.

The comparison of tissue-resident macrophage-specific genes and TAM-specific genes has resulted in the identification of a group of six genes (*MS4A6A, LILRB5, CD209, CD163, FOLR2, C1QC*) which are also preferentially/exclusively expressed by macrophages with an anti-inflammatory/reparative polarization (included within the “anti-inflammatory” gene set). The proteins encoded by these six genes participate in either pathogen recognition (*CD209, CD163, C1QC*) or in modulation of inflammatory responses (*MS4A6A, LILRB5*). By contrast, and apart from its folate-binding ability, FRβ does not appear to fit within any of these two classes, although it modulates macrophage adhesion to collagen through association to the CD11b/CD18 integrin [92]. As a glycosyl phosphatidylinositol (GPI)-anchored protein, FRβ’s potential to exert immunoregulatory actions would be indirect. By contrast, the cellular distribution, structure and recycling behavior of FRβ [85] somewhat resembles that of CD14, a crucial regulator of TLR4 ligand binding, endocytosis and TLR4-initiated signaling from endosomes [93,94]. We speculate that FRβ might exhibit a function similar to CD14, which acts both as a pattern-recognition receptor that binds directly to LPS and a co-receptor for several TLRs [95]. FRβ has a very high affinity for folic acid and folates (Kd ~ 0.1–1nM), but mammals do not synthesize folate and are dependent on other sources. Diet or dietary supplements are not the only sources of folate, as several bacteria in the gastrointestinal tract can synthesize B vitamins, including folates (e.g., *Lactococcus lactis*, *Bifidobacterium adolescentis*) [96,97]. The macrophage-specific expression of FRβ described in this report, and the fact that folic acid is produced by numerous bacterial species [96,97], have led us to hypothesize that FRβ acts as a receptor or co-receptor for recognition of bacterial microbiota. If so, gut macrophages could detect high concentrations of folate through FRβ as a mechanism to control bacterial overgrowth through signaling or by phagocytosis, which would allow the microbiota homeostasis to be restored/maintained by a folate-dependent quorum sensing-like mechanism. Whether this mechanism contributes to the interplay between TAM and human microbiota in cancer [98] deserves further investigation. In any event, the hypothesis that FRβ is a sensor for adjusting macrophage effector functions to extracellular folic acid levels is fully compatible with the findings reported in the present manuscript, namely, that FRβ marks tissue-resident macrophages and macrophages within inflamed tissues, and that its expression correlates and is dependent on the expression of the PU.1 transcription factor.
